# Health Literacy‐Focused Communication Training for Primary Healthcare Providers Working With Older Adults: A Co‐Designed Prototype

**DOI:** 10.1111/hex.70590

**Published:** 2026-02-08

**Authors:** Lesley O'Brien, Michael Lawless, Louise Townsin, Peter Mills, David Ta, Justin Beilby, Glenn Errington, Rachel Ambagtsheer

**Affiliations:** ^1^ Centre for Public Health Equity and Human Flourishing Torrens University Australia Adelaide South Australia Australia; ^2^ Caring Futures Institute Flinders University, College of Nursing and Health Sciences Adelaide South Australia Australia; ^3^ Centre for Healthy Sustainable Development Torrens University Australia Adelaide South Australia Australia; ^4^ Adelaide Rural Clinical School University of Adelaide Adelaide South Australia Australia; ^5^ College of Science and Engineering Flinders University of South Australia Adelaide South Australia Australia

**Keywords:** co‐design, communication training, health literacy, older adults, patient‐centred care, primary healthcare, provider education

## Abstract

**Introduction:**

Low health literacy among older adults living in the community contributes to poor health outcomes. While communication training exists for specific conditions (e.g., hypertension), there is limited evidence on programs that enhance health literacy competencies among primary healthcare providers working with older adults. This study aims to co‐design a health literacy‐focused communication training program for general practitioners, practice nurses and allied health professionals in primary care.

**Methods:**

This study drew on an Experience‐Based Co‐Design approach; three online workshops were conducted over 2 months with a participant group of adults aged 50+ years and primary healthcare professionals, facilitated by a multi‐disciplinary research team. Activities involved a needs assessment informed by a prior scoping review, structured ideation and post‐workshop surveys to prioritise communication competencies, training outcomes and delivery preferences. Competencies were synthesised into draft program modules, and participants reflected on the co‐design experience.

**Results:**

Seven participants identified and prioritised nine core communication competencies spanning knowledge, skills and attitudes. The competencies were grouped into three‐modules, deliverable by a mix of online self‐paced subjects and in‐person simulations. Evaluation surveys and qualitative feedback showed positive participant engagement in the sessions. The co‐design process was refined iteratively to improve clarity and structure.

**Conclusion:**

This structured yet flexible co‐design process resulted in a final training program that integrates real‐world needs with pedagogical frameworks and aligns with evidence from prior training interventions. There is potential for implementation in primary healthcare provider training environments.

**Patient or Public Contribution:**

Older adult consumers contributed lived experience to the co‐design workshops, interpreting findings and shaping the training prototype. This manuscript was reviewed by a consumer.

## 1 Introduction

Low health literacy (HL) is prevalent among older adults, correlating with poorer health outcomes, such as lower uptake of preventive services [1, 2, 3, 4, 5]. Nearly half of adults aged 50 years and older have limited HL, with estimates ranging from 40% to 60% across OECD countries [6]. HL (i.e., the ability to access, understand, evaluate and apply health information) is critical to effective healthcare provision [7, 8], particularly for adults over 50 years of age who may face cognitive changes, sensory impairments and the challenges of polypharmacy [9]. Adults aged 50 years and older are a key target group for HL initiatives due to their higher likelihood of managing chronic conditions, increasing involvement in healthcare decision‐making and growing need for effective self‐care strategies [10, 11]. Chronic conditions and cognitive shifts that may affect HL often emerge well before the conventional older adult age of 65 years, and age is a key factor associated with lower HL [9, 12].

The contexts in which HL develops are shaped by complex health systems, social determinants and the dynamics of the care relationship [13, 14]. Within these contexts, primary healthcare providers (PHCPs) play a key role in improving patient HL [15, 16, 17] through the process of health communication. Health communication (i.e., the use of evidence‐informed communication strategies to promote health and improve HL) can involve interpersonal communication, mass communication and public health campaigns [18]. Interpersonal communication is the exchange of information, ideas and emotions between two or more people, can occur through verbal and nonverbal cues [19] and is the most relevant medium for PHCPs in the context of a healthcare consultation. PHCP communication strategies (e.g., using plain language, teach‐back and shared decision‐making) through dyadic interactive (i.e., two‐way synchronous) communication during health service delivery can mitigate literacy gaps and improve patient health outcomes [20, 21]. The challenge for PHCPs, who frequently lack opportunities to establish long‐term, trusting relationships with their patients [22], is the need to quickly gauge patients' HL levels relying on their professional experience and judgement in short consultation times, as this is a critical aspect of self‐care capability [23, 24, 25, 26], and tailor communication and care provision approaches accordingly [27, 28]. HL‐focused communication for PHCPs is multi‐faceted; made up of the providers' own HL ability and their knowledge, attitudes and skills to strengthen and support HL in others (e.g., patients and their relatives) [29].

HL‐focused provider communication training interventions aim to improve patient HL, progressing from functional (i.e., basic reading and writing skills) to communicative/interactive (i.e., advanced cognitive and social skills for exchange of information) and ultimately to critical HL abilities, whereby more advanced cognitive and social skills can be used to critically analyse information and exercise greater control over life situations [30]. While improving PHCP HL and communication skills can enhance outcomes and reduce costs, training interventions to date have a number of limitations [5]. Although many have been collaboratively developed with stakeholders, incorporate diverse methods and modalities, and report positive impacts on providers' HL, self‐efficacy and communication skills [31], they typically focus on narrow contexts (e.g., telephone consultations) or outcomes (e.g., palliative care communication). Few define or address the core communication competencies relevant to interactions with older adult patients more broadly. Core communication competencies are structured, foundational abilities (i.e., knowledge, skills and attitudes), such as initiating and closing consultations, gathering and explaining information, building rapport, active listening and adapting communication, that are exemplified in models like the Calgary–Cambridge framework, which outlines the structured process of clinical communication relevant across diverse settings [32].

Sets of core communication competencies for PHCPs are described in the literature, yet gaps exist in standardisation with variations depending on the country, institution or professional body [33]. These competencies are often framed within clinical or professional education standards, or tailored to specific contexts, including HL‐sensitive studies, or found in geriatric research [34, 35, 36]. The role of communication in safety and quality of care is recognised [37, 38, 39] and standards exist to crosscut such as communication across different modes (e.g., in‐person, telehealth). However, most training interventions have focused on practical communication skills rather than aligning content with structured competency frameworks that incorporate knowledge, skills and attitudes [31, 40, 41]. While some programs incorporated elements like shared decision‐making and patient‐centred communication, they were rarely framed within recognised models (e.g., Calgary–Cambridge Guide, Health Belief Model and Shared Decision Making). Few studies evaluated provider self‐efficacy across domains such as linguistic and nonverbal communication, and competencies related to HL assessment, cross‐cultural communication and supporting autonomy in older adults remain underrepresented. Overall, prior training interventions lack integration of competency‐based frameworks to guide content, delivery and evaluation. This represents a gap in aligning training with the broader primary care communication standards.

The noted limitations of existing training interventions, particularly the lack of co‐design with both PHCPs and consumers, suggest limited adaptability to diverse healthcare settings and the complex needs of older adults [42, 43, 44, 45]. These needs include cognitive and sensory impairments, cultural diversity, polypharmacy and multimorbidity [46, 47]. Locally responsive, co‐designed interventions with a focus on general interpersonal communication principles and continued improvement through embedded evaluation are needed [48]. In summary, while numerous studies have reviewed the importance and benefits of improving HL among the general population, few communication‐focused training interventions have targeted PHCPs working with community‐dwelling older adults in primary care [31, 49]. This study addresses that gap by identifying and prioritising core PHCP communication competencies, with a focus on interpersonal communication, that can improve HL‐focused outcomes (i.e., a measurable improvement in accessing, understanding or using health information) in adults 50+ years of age [29].

## 2 Methods

### 2.1 Design

An Experience‐Based Co‐Design (EBCD) approach was applied to collaboratively develop a communication training for PHCPs with consumers and providers. EBCD is a participatory, narrative‐driven approach that typically involves service users and staff in improving healthcare services [50]. The approach was adapted for the online setting of this study and time constraints, drawing on the EBCD Australia Toolkit [51] and previous successful adaptations in healthcare intervention design [50, 52, 53].

**Figure 1:**
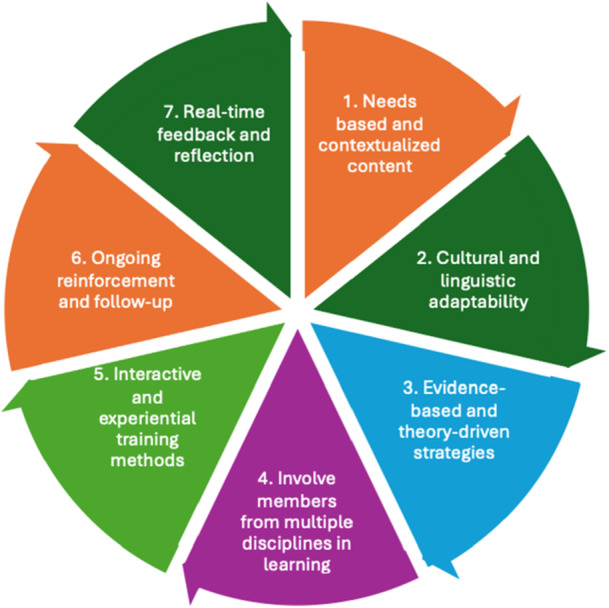
Seven key factors in developing provider training for positive outcomes from ideation to implementation.

This paper reports on the development stage of EBCD and reports on the co‐design process (Workshops 1–3) and its output (training prototype). It is informed by prior work (i.e., scoping review to understand existing interventions) and leads toward a future implementation study to test and refine the intervention in a primary healthcare setting [54]. This structure supported a training intervention grounded in evidence and shaped by lived and professional experiences in real‐world contexts. Each workshop followed a detailed facilitation plan and was iteratively adapted based on participant feedback. Discussions focused on identifying communication barriers, prioritising learning needs and shaping training delivery formats. This study was informed by a previous review that identified 18 PHCP training interventions (2010–2024), many of which used collaborative processes with stakeholders in design and/or facilitation [31]. These interventions demonstrated generally positive outcomes in provider HL, self‐efficacy and communication skills. A range of principles guided the co‐design and training program development, including the EBCD framework, the Knowledge Skills Attitudes (KSA) framework [40], process models [55, 56] applied flexibly to accommodate iterative provider and consumer input, and key factors for effective provider training (Figure 1) [31]. The KSA framework, used to structure the co‐design, emphasises the integration of practical skills with knowledge and attitudes, particularly relevant in dynamic healthcare contexts like HL [40]. It also supports competency evaluation and curriculum development.

Three co‐design workshops were conducted over 6 weeks, with structured online Zoom workshops involving PHCPs and older adults. The process was evaluated using triangulated data from post‐workshop surveys, confirming the approach was meaningful and acceptable to participants [57].

### 2.2 Participants and Recruitment

Flyers and invitations were distributed via direct email, researcher networks (e.g., the Australian Association of Gerontology member forum) and social media channels. Participants responded by email with expressions of interest to voluntarily participate in the study. All participants were informed of the reasons for the study and gave written informed consent by signing a Participant Information and Consent Form. Participants were included if they were available to attend three workshops online in English and complete online surveys. To protect privacy, each participant was assigned a deidentified ID before completing demographic and evaluation surveys. Participants were offered an honorarium gift card for their time [58]. The ethical aspects of this research project have been approved by the Human Research Ethics Committee (Number 0384). This project was carried out according to the National Statement on Ethical Conduct in Human Research (2023).

After initially aiming to recruit 8–12 participants, we recruited 4 PHCPs (i.e., general practitioners, practice nurses) and 3 consumers over 50 years of age to ensure a mix of professional and lived experience perspectives. The relatively small sample size in this study aligns with the ‘small co‐design team’ stage reported in previous EBCD work, which involves convening groups of between 4 and 20 participants including both service users (patients) and service providers (staff) to work on specific aims/product development [50, 59, 60]. The group composition was intentionally balanced to capture both provider insights into communication practice and consumer reflections on receiving care, recognising that each group brings different forms of expertise. This structure likely contributed to the practical relevance and contextual grounding of the training prototype, although it also raises questions about how such dynamics might shift in larger or more demographically diverse cohorts. Consumer characteristics facilitated engagement but may not represent the perspectives of older adults with limited HL.

All seven participants remained engaged throughout the study period, though not all attended every workshop due to work or travel commitments, with typically one or two absences per session. Participants actively requested updates on sessions they missed and collaboratively selected subsequent workshop dates to maximise attendance.

### 2.3 Workshop Process

**Figure 2:**
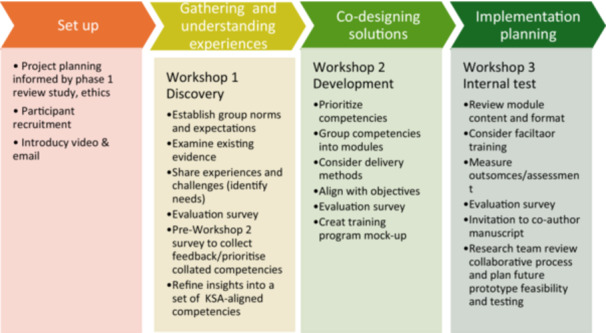
Co‐design workshop series.

The co‐design process was guided by the six stages of EBCD, structured across a series of three 1‐h online Zoom workshops (Figure 2). No non‐participants were present during the workshops.

#### 2.3.1 Stage 1 Set‐Up

Prior to the workshops, an introductory video and PowerPoint presentation summarising relevant findings from our earlier scoping review were emailed to participants to establish a shared understanding of key concepts (e.g., HL, training objectives) [61]. This stage also included developing an approach to workshop facilitation.

#### 2.3.2 Stage 2 Gathering Experiences

Workshop 1 focused on surfacing lived and professional experiences with HL‐related communication. Activities included group discussion of existing training barriers, exploration of interpersonal communication challenges, and critical reflection on participants' own encounters with communication in primary care [62]. The discussion was prompted by visual summaries of prior research (e.g., 25‐item communication checklist). Participants identified broad training needs, such as managing limited time, recognising low HL and addressing unconscious bias. This session led to the introduction of the KSA framework as a tool for framing competencies.

#### 2.3.3 Stage 3 Understanding the Experience

Between Workshops 1 and 2, participants completed a survey to prioritise 17 potential competencies drawn from three sources: our scoping review, the literature and insights from Workshop 1. The competencies were labelled as knowledge, skills or attitudes, and participants were encouraged to suggest refinements. This stage synthesised diverse inputs to form a shared understanding of which competencies mattered most and why. Participant evaluation of Workshop 1 feedback indicated a need for clearer structure and stronger scaffolding, prompting revisions to materials and facilitation in subsequent sessions.

#### 2.3.4 Stage 4 Improving the Experience

In Workshop 2, participants refined the content and structure of the training intervention. Using survey results, the research team facilitated a discussion to group high‐priority competencies into three proposed modules, identifying overlaps, gaps and points of confusion (e.g., empathy as both skill and attitude). The group also explored delivery preferences (e.g., online vs. in‐person), drawing on concrete examples from existing training programs [63]. Participants highlighted the importance of interactive formats, such as role play and reflective practice.

Workshop 3 focused on prototype refinement. Participants reviewed a mock‐up of the proposed modules, discussed learning outcomes and considered practical implementation issues, including facilitator requirements and assessment methods. Input from Workshop 2 was integrated into this draft. Participants were invited to co‐author the manuscript, in line with the final EBCD principle of shared ownership.

#### 2.3.5 Stage 5 Implementation Planning

Workshop 3 initiated preliminary planning discussions. Participants considered delivery formats, resource implications and integration into primary healthcare training systems. Participant feedback was used to inform proposals for pilot testing and evaluation.

#### 2.3.6 Stage 6 Celebration and Review

Participants were acknowledged and thanked at the conclusion of the final workshop. An invitation to join the authorship team was extended to interested participants. Final feedback was collected through surveys and informal emails. It was noted that formal survey response rates declined yet high informal input was sustained, and it reflected shared achievement and respect for the co‐design process.

The research team consisting of three females and a male, from two different universities met fortnightly to plan logistics and reflect on how individual disciplinary perspectives and facilitation styles might influence the co‐design group dynamic/progress. Drawing on established co‐design facilitation principles, the meetings were used to sketch out run sheets, revisit insights from earlier sessions and refine the structure of upcoming workshops. This meant tweaking discussion prompts, shifting roles to match the flow of conversation or rethinking how best to introduce complex material in ways that felt accessible without oversimplifying. Anticipating facilitation challenges, such as managing quieter voices or balancing the input of professional and consumer participants, was an ongoing part of this work. To keep the space as equitable as possible, we rotated facilitation responsibilities while ensuring continuity through one lead facilitator (L.O.), in line with co‐design recommendations for minimising hierarchy and encouraging inclusive participation.

### 2.4 Data Collection and Analysis

Workshop discussions were recorded and transcribed, with thematic analysis of training program features conducted by the research team (R.A., L.O., L.T. and M.L.). Analysis focused on identifying communication competencies, pedagogical preferences and illustrative examples of effective practice from workshop discussions. Thematic analysis was conducted reflexively, using both inductive coding from participant narratives and deductive analysis guided by the KSA framework and EBCD principles. One researcher (L.O.) coded data, with themes refined iteratively through team discussion and validation against workshop outputs. Participants did not review the transcripts for accuracy. Quantitative data included pre‐/post‐workshop surveys (e.g., Likert items on perceived influence), participant demographics and ranking of communication competencies according to priority and delivery method. Survey data were analysed descriptively using SPSS (L.O.). For competency prioritisation, items ≥ 60% agreement were considered consensus based on established consensus methodology for small expert groups [64].

The co‐design process was internally evaluated after each workshop by comparing workshop outcomes (e.g., participant attendance, researcher field notes and activity outputs) with the planned agenda in post‐session debrief meetings of the research team. A summary report of participant evaluation was tabled at research team workshop preparation meetings and critically discussed along with the sharing of research team field notes. Points of concern were addressed in the next workshop. Additionally, qualitative data were collected and analysed from participant feedback to open‐ended evaluation survey questions (L.O.). Responses to the evaluation survey (Supporting Information S1) were extracted and collated with quantitative scores (i.e., 1 strongly disagree to 5 strongly agree) and qualitative feedback themed (e.g., pace of workshop). Qualitative data from surveys were thematically analysed and grouped into clusters of related meaning, which were iteratively refined into broader categories. This study is reported in line with the Consolidated Criteria for Reporting Qualitative Research (COREQ) 32‐item checklist for focus groups (Supporting Information S4) [65].

## 3 Results

### 3.1 Participant Characteristics

Seven participants took part (43% female), including three consumers (patients and carers) and four healthcare providers (one practice nurse and three doctors) from four Australian states (Table 1). Two consumers had gerontology experience: one as an academic and one as a patient health educator. Participants brought diverse experiences, ranging from academic research to involvement in clinical guideline consultation. One doctor was working in an aged care (i.e., residential nursing home) setting. Ages ranged from under 50 (*n* = 1), 50–64 (*n* = 3) and 65–84 years (*n* = 3). Most participants were male (*n* = 4), all had English as their main language spoken and most participants were born in Australia, with one born in the United States.

**Table 1:** Participant characteristics.

Characteristics	*N* (%)
Role	
Provider	4 (57)
Consumer	3 (43)
Age	
35–49	1 (14)
50–64	3 (43)
65–84	3 (43)
Gender	
Male	4 (57)
Female	3 (43)
ATSI	
No	7 (100)
Yes	0
COB	
Australia	6 (86)
United States	1 (14)
Main language	
English	7 (100)
Previous experience in research	
Yes	7 (100)
No	0

Abbreviations: ATSI, Aboriginal or Torres Strait Islander; COB, country of birth.

### 3.2 Final Needs‐Based Training Prototype

The final training prototype consisted of three modules with nine core communication competencies and three sub‐competencies, refined iteratively through survey feedback and workshop discussions. In Workshop 2, participants reviewed a set of 17 proposed communication competencies drawn from published training interventions, previous scoping review findings, and themes raised during Workshop 1 (Supporting Information S2). The KSA framework was used flexibly as an organising tool rather than a prescriptive model.

Pre‐Workshop 2 survey ranking of 17 identified competencies found 80% of participants agreed that *Introduction to geriatrics*, *Interpersonal communication principles* and *Adjusting medical jargon* could be taught and learnt effectively online. Competencies that participants felt required in‐person role‐play or simulation practice, such as *Using appropriate methods to assess understanding* and *Detecting patient information withholding*, also received 80% agreement on ranking and delivery method (i.e., online, in‐person). Consensus was defined as ≥ 60% agreement, with competencies below this threshold excluded from the final set [64]. The result was nine core competencies, including three sub‐competencies nested within modules, considered essential for effective PHCPs–older adult communication (Table 2).

**Table 2:** Final training prototype.

Module	Title/focus	Key themes/content	Learning resources/tasks/assessments	Suggested delivery mode/duration
1	Foundations of communication/building foundations	Ageing and its impact on communication Interpersonal principles Recognising limited HL	Short video lectures Interactive quizzes Animated case vignettes Self‐assessment checklist	Online self‐paced/3 h
2	The art of patient‐centred dialogue/enhancing communication	Creating supportive dialogue Culturally responsive communication Trauma‐informed approach Mitigating unconscious bias	Video demonstrations Structured interview guide Peer role‐play Reflective journal Standardised patient (SP) objective structured consultations	Online pre‐work and face‐to‐face workshop/4 h
3	Mastering complex clinical conversations/strengthening relationships and trust	Handling sensitive topics Advanced questioning and listening Addressing misinformation	Live simulation with SPs Checklist‐based feedback Case‐study debrief Self‐reflection worksheet	In‐person intensive/4 h

*Note:* See Supplementary S3 for training modules mock‐up.

Participants' preferences, priorities and feedback shaped the structure of the three‐module training (Table 2). Findings from each workshop were validated by participants in the following workshop. Participants emphasised the importance of training formats that align with real‐world practice constraints. Online modules were considered essential for foundational knowledge, while in‐person elements were necessary for building nuanced skills.

The focus of **Module 1: Foundations of Communication** is how ageing may influence provider–patient interactions and the principles of interpersonal communication. It emphasises the importance of recognising limited HL early and tailoring responses accordingly. Online, self‐paced resources were preferred for this introductory content to allow flexible access. **Module 2: The Art of Patient‐Centred Dialogue** responded to participants' concerns about trust and relational dynamics. This module centres on active listening, trauma‐informed care and mitigating unconscious bias. A blended format of pre‐reading, followed by facilitated in‐person workshops, was co‐developed and incorporates role play, reflective journaling, and simulated conversations. **Module 3: Mastering Complex Clinical Conversations** is designed to address difficult topics like managing misinformation, supporting autonomy and engaging family members. Participants supported using live simulations and structured feedback to enable PHCPs to practise and reflect on challenging interactions.

Participants' discussions shaped the conceptualisation of each module. For Module 1, providers emphasised the need to recognise limited HL early in consultations. In Module 2, participants discussed whether empathy was a teachable skill or an inherent attitude, ultimately agreeing that it required both self‐awareness and practised techniques like reflective listening. Consumer participants stressed the importance of meeting patients where they are rather than where providers might expect them to be. For Module 3, regarding misinformation, participants agreed that correction should begin with understanding the emotional or cultural meaning behind beliefs. Family involvement was seen as both a resource and a challenge, requiring clear boundaries. Participants identified culturally responsive communication as essential, particularly recognising that health beliefs and family involvement vary across cultural backgrounds and require tailored approaches rather than standardised scripts.

Participants also recommended recognition for continued professional development (CPD) to support uptake. Overall, the modular structure was intended for integration across varied PHCP training settings (e.g., universities, clinics) for both pre‐professional and professional continued learning.

### 3.3 Co‐Design Process

Key themes stabilised from the co‐design process were achieved after three evaluation surveys were completed by participants and audio transcripts of the workshops were analysed (Table 3). Survey participation declined across sessions (Survey 1: *n* = 6; Survey 2: *n* = 4; Survey 3: *n* = 2), with only Survey 1 having sufficient responses for formal reporting. Participants did not state reasons for declining survey completion yet sustained feedback via email and during workshop discussions suggests a preference for conversational over structured input. Survey 1 results indicated that participants agreed/strongly agreed the workshops held together with healthcare providers and consumers were useful (3/6, 50%), interesting (3/6, 50%) and feasible (5/6, 83%) (Supporting Information S1). Most respondents valued the exchange of diverse perspectives (Table 3). Most participants (4/6, 67%) felt comfortable sharing their ideas in the first workshop, felt they were considered and that the session was clear and well‐paced, adding that it generated valuable and creative ideas. Although most of the participants were not satisfied/unsure that we designed collectively as a group in the first workshop. In total, 83% of participants from the first workshop evaluation survey agreed that the facilitators guided discussion and activities well. Key areas for improvement from open‐ended questions on what participants found most valuable and what could be improved included clearer framing, stronger preparatory materials and more flexible scheduling.

**Table 3:** Themes identified from the co‐design process.

Theme	Description	Illustrative quote	Workshops
Collaborative learning and diverse perspectives	Participants valued hearing others' perspectives and active listening	‘Hearing the thoughts of the other participants’; ‘Listening to the different viewpoints of the attendees’	W1, W2
Open and safe space for contribution	Freedom for everyone to contribute without being shut down fostered engagement	‘The ability for everyone to freely contribute without being shut down’	W3
Motivation to address complex topics	Group showed willingness to tackle broad, complex health‐literacy issues.	‘The willingness to tackle many or all of the most pressing topics within the broad spectrum of this study’	W1
Need for clearer structure and goals	Participants requested upfront outlines, frameworks and clarity of objectives for each session	‘To have an outline given at the beginning’; ‘Structure, goals and leadership. Clarity of objectives’	W1, W2, W3
Time limitations	One‐hour workshops felt rushed and limited depth of discussion	‘There doesn't seem to be enough time’	W2
Provide preparatory materials	Pre‐reading or materials before workshops could streamline decision‐making	‘Doing pre‐reading and ideas before workshop so we can make decisions in the short one hour meeting’	W1
Declining engagement in the evaluation surveys yet consistent attendance in workshops	Noted drop‐off in participant completion of evaluation surveys across sessions	‘Completion of surveys seems to have fallen away in lieu of vocal and emailed feedback’	W3

## 4 Discussion

This study focused on the co‐design stage and developed an HL‐focused communication training prototype for PHCPs who work with older adults. What emerged was a program with three modules shaped by the lived experiences and practical concerns of both healthcare professionals and older adult consumers. The study presents a prototype that reflects a shared understanding of what could be operationalised in primary healthcare settings [66].

The competencies identified in this study (e.g., empathy, active listening and cultural sensitivity) mirror those frequently cited in health communication literature [1, 20, 28]. However, their prioritisation suggests what participants felt was missing or under‐emphasised in current training offerings [52]. Many of the insights garnered from the workshops reiterated themes from earlier studies [1, 20, 28, 29, 50, 52, 67, 68], particularly around the need for relational skills to sit alongside technical knowledge. This informed the structure of the topics for each training module. Opportunities to practise, reflect and receive feedback, whether through role play or discussion, were considered essential. One undercurrent throughout the workshops was the challenge of designing for complexity without losing clarity. The structure of discussions was supported by the application of the KSA framework and loosely drawing on established process models, including experiential learning principles [56] and the iterative stages of instructional design [55].

The process by which this set of communication competencies was developed and categorised into a tangible research output through this study represents a distinctive contribution. Unlike more conventional, top‐down approaches where training content is shaped by academic or institutional priorities, this training program emerged through direct collaboration with older adults and PHCPs. The co‐design method placed emphasis on negotiation, responsiveness and shared decision‐making to refine the material and reframe an approach to curriculum design [50, 52]. While participation in surveys dropped by the third workshop, the feedback that continued via email and direct discussion suggests the sessions were engaging and meaningful. Several participants remarked on the unexpected value of hearing perspectives from others whether as a provider listening to patient stories or as a consumer learning about the pressures of general practice. These comments reflect a shift in understanding that comes from working through problems together [5, 52].

Early sessions felt fragmented to some participants, yet iterative changes such as refining run sheets and clarifying objectives helped build structure and participant confidence in the process. By the final session, participants reported greater ease in contributing, highlighting the value of group learning and ideation. Several participants appreciated the opportunity to explore the complexities of HL, rather than resorting to oversimplified solutions. Across all three workshops, the need for concise framing and well‐paced content was consistently raised.

Co‐design lends itself to the development of training interventions through iterative design with stakeholders as it is narrative‐driven and emotion/felt sense impact is taken into account as well as efficiency and clinical outcomes [54]. The focus on real‐world needs and influencing environmental factors can result in improved relevancy and support implementation of the training intervention output while capturing the nuances of interpersonal communication in professional care settings [45, 47, 48, 50]. However, few are designed with future student input [31, 69]. This hinders replication and the specific considerations for involving participants such as older adults with health professionals in the design process, where attention to power dynamics, accessibility and the lived experiences of both groups is required to ensure meaningful engagement and sustainable outcomes [70].

Certain features of the online co‐design workshop format appeared to support participation. For example, keeping cameras on was perceived to enhance connection and improve conversational flow. The facilitation approach, grounded in structured run sheets, debriefs and iterative adaptation, was made feasible through the planning of adequate time, funding and resourcing. Across sessions, real‐time communications and emailed reflections helped maintain an inclusive and participatory tone. The resulting training prototype is a product of shared ownership, designed through lived experience and negotiated meaning.

Study strengths include the team's expertise (i.e., gerontology, health services research, training design, co‐design and knowledge translation) and active general practitioner involvement, which enhances practical relevance and contextual grounding. Limitations include the small, demographically narrow sample, brief workshop duration and focus on perceived rather than tested outcomes. While the final group of seven participants enabled rich discussion and consensus, all three consumer participants were highly educated, two with backgrounds in health. This facilitated sophisticated engagement but may not represent the perspectives of older adults with limited HL. Future work should engage more diverse cohorts across the HL spectrum using adapted methods such as one‐on‐one interviews or community partnerships.

## 5 Conclusion

The participatory co‐design methodology applied in this study, including topical design frameworks (i.e., KSA, process models), contributes an HL‐focused communication training prototype tailored to general PHCP interpersonal interactions with older adults. To the authors' knowledge, this is the study to identify HL‐focused communication competencies through needs‐based co‐design into a PHCP training prototype aimed at improving HL in older adult patients. PHCPs and patients can both benefit from training focused on provider communication competencies in terms of self‐efficacy and improved HL. Improved provider–patient communication has the potential to positively impact patient health outcomes, reduce medical errors, lead to better adherence to treatment plans and over all reduced healthcare costs. In addition, there is potential for increased PHCP job satisfaction and improved team collaboration due to enhancing individual critical interpersonal and interactive communication skills. Online co‐design workshops provide a low‐cost, accessible way to hold sessions when face‐to‐face is not possible. Although resource‐intensive, this well‐structured, focused co‐design approach offers a promising way to improve health services.

A program that teaches core interpersonal communication competencies, adapts to different healthcare and cultural contexts and is refined through ongoing evaluation shows promise as a sustainable solution.

## Author Contributions

**Lesley O'Brien:** study design, methodology, data analysis, review and editing. **Michael Lawless:** study design, methodology, supervision, funding, review and editing. **Louise Townsin:** study design, methodology, supervision, review and editing. **Peter Mills:** review and editing. **David Ta:** review and editing. **Justin Beilby:** review and editing. **Glenn Errington:** review and editing. **Rachel Ambagtsheer:** study design, methodology, supervision, review and editing. All authors read and approved the final manuscript.

## Funding

The authors received no specific funding for this work.

## Ethics Statement

The ethical aspects of this research project have been approved by the Human Research Ethics Committee (HREC) of Torrens University Australia (#0384). This project was carried out according to the National Statement on Ethical Conduct in Human Research (2023).

## Conflicts of Interest

The authors declare no conflicts of interest.

## Supporting information


**S1:** Demographic and evaluation survey questions.

**S2:** Pre co‐ design session 2 survey.

**S3:** Training modules mock up.

**S4:** Consolidated Criteria for Reporting Qualitative Research (COREQ): 32‐ item checklist.

## Data Availability

The data that support the findings of this study are available in the supplementary material of this article. Other data are not publicly available due to privacy or ethical restrictions.
