# Supplementary material for: Health Literacy‐Focused Communication Training for Primary Healthcare Providers Working With Older Adults: A Co‐Designed Prototype
Source: Health Expect. 2026 Feb 8;29(1):e70590. doi: 10.1111/hex.70590 (PMC12883676; doi:10.1111/hex.70590)
Supplement: Supplementary file 1 — S1: Demographic and evaluation survey questions. [file HEX-29-e70590-s002.pdf]

Intro and ID

Evaluation of the Provider Communication Training Co-design workshop

Thank you for joining us in the recent co-design workshop. Please help us evaluate the session by answering the questions in this short survey.

Please enter your participant ID

Demographics

Are you a primary health care provider, patient or carer? If yes, please elaborate

How old are you in years?

35-49

50-64

65-84

85+

What is your gender?

Male

Female

Non-binary

Prefer not to say

Are you of Aboriginal or Torres Strait Islander origin?

Yes

No

Prefer not to say

In what country were you born?

What is your main language spoken at home?

Process and engagement

Have you been involved in research before?

Yes (please detail below)

Maybe

No

I found the online workshop held together with health care providers and consumers...

	Strongly disagree	Disagree	Neither agree nor disagree	Agree	Strongly agree
Useful	○	○	○	○	○
Interesting	○	○	○	○	○

	Strongly disagree	Disagree	Neither agree nor disagree	Agree	Strongly agree
Feasible	○	○	○	○	○

Please rate for each of the statements

	Strongly disagree	Disagree	Neither agree nor disagree	Agree	Stror agre
I felt comfortable sharing my ideas and opinions openly.	○	○	○	○	○
We designed as a collective group.	○	○	○	○	○
My ideas were considered and integrated into the overall discussion.	○	○	○	○	○
The workshop facilitator(s) guided the discussion and activities well.	○	○	○	○	○
The workshop structure was clear and well-paced.	○	○	○	○	○

	Strongly disagree	Disagree	Neither agree nor disagree	Agree	Stror agree
I had opportunity to contribute my ideas and perspectives.	○	○	○	○	○
I am satisfied with the overall co-design workshop experience.	○	○	○	○	○

Did the workshop generate valuable and creative ideas?

Yes

No

Unsure

What did you find most valuable about the workshop?

What could be improved in future co-design workshops?

Powered by Qualtrics
