# Supplementary figures and images for: Health Literacy‐Focused Communication Training for Primary Healthcare Providers Working With Older Adults: A Co‐Designed Prototype
Source: Health Expect. 2026 Feb 8;29(1):e70590. doi: 10.1111/hex.70590 (PMC12883676; doi:10.1111/hex.70590)

**S3 Training modules mock up**


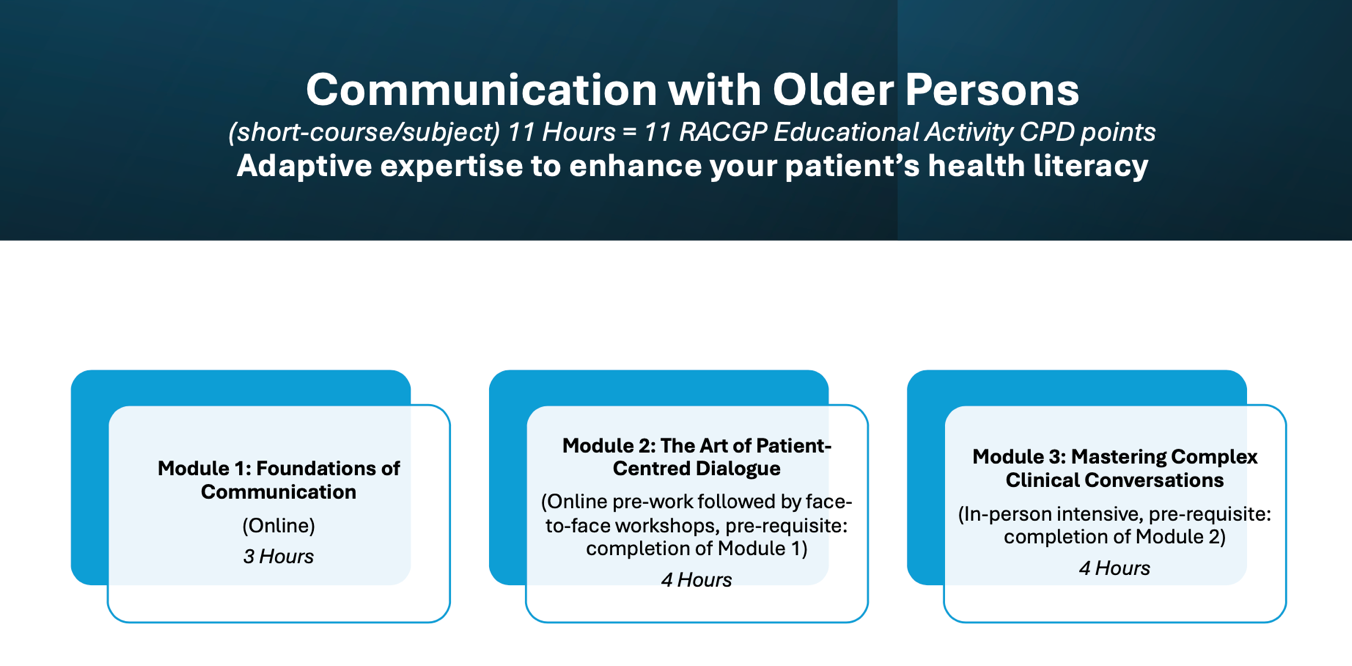


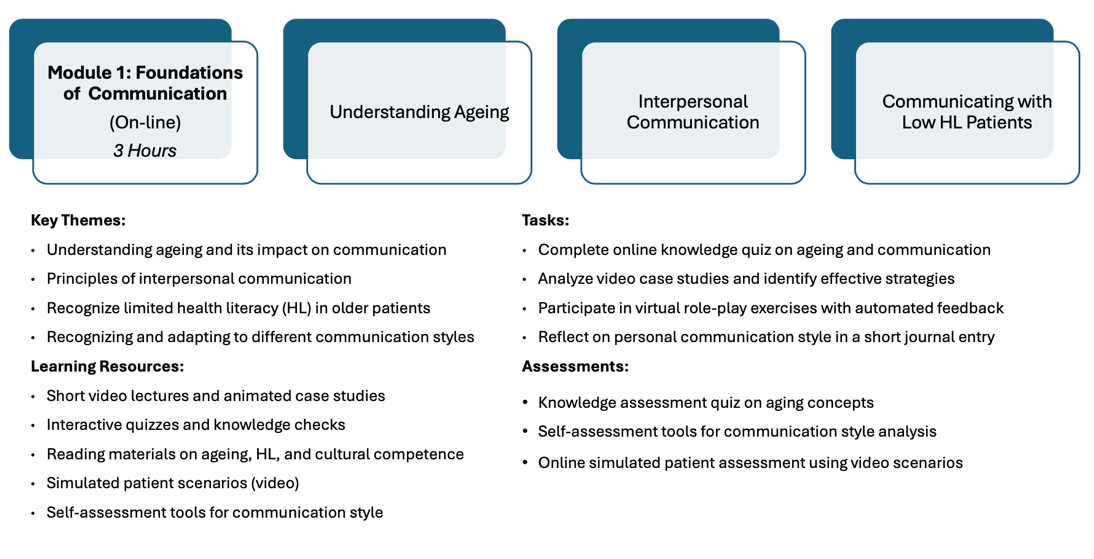


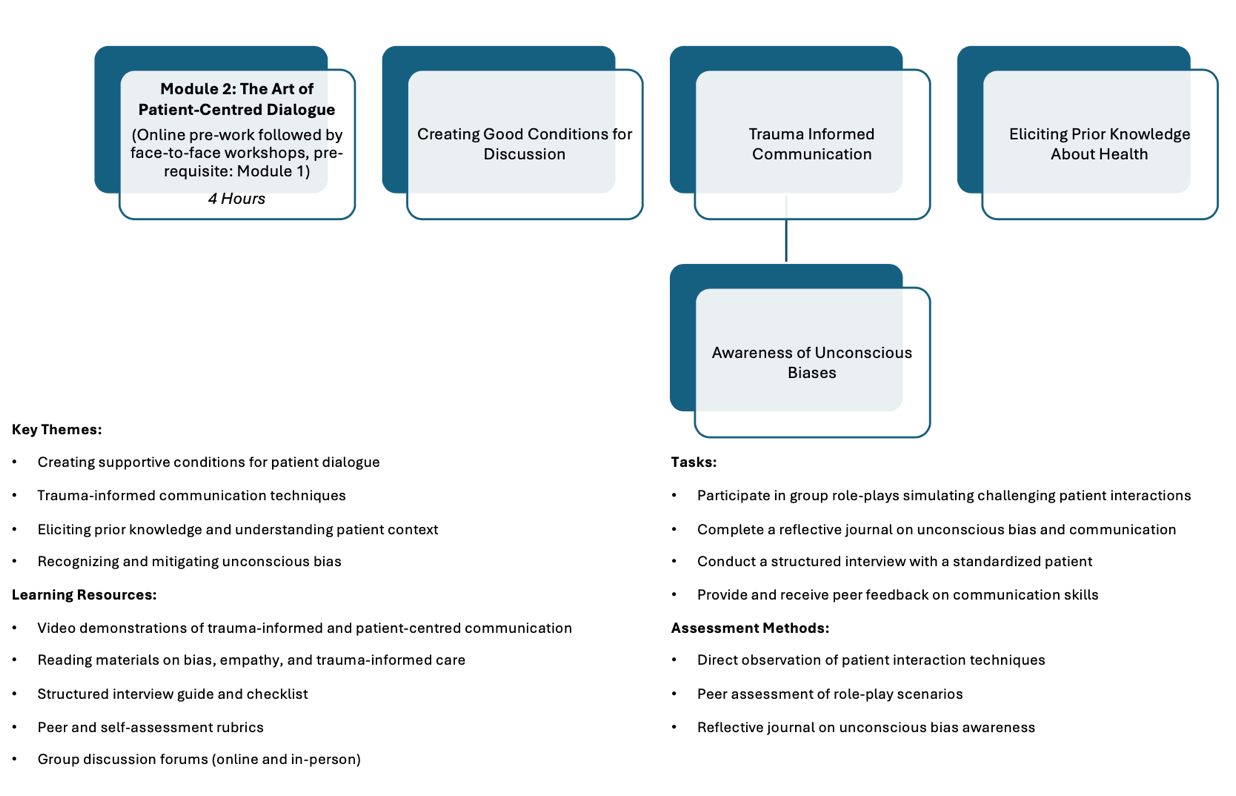


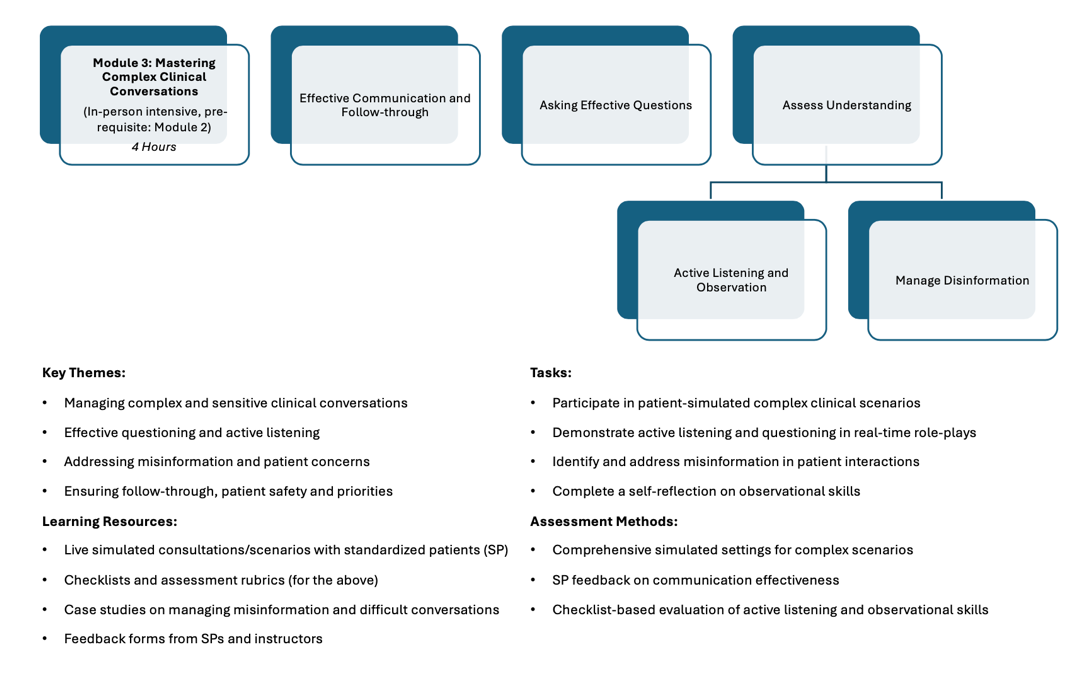

Supplement: Supplementary file 3 — S3: Training modules mock up. [file HEX-29-e70590-s003.docx]
