# Supplementary material for: Health Literacy‐Focused Communication Training for Primary Healthcare Providers Working With Older Adults: A Co‐Designed Prototype
Source: Health Expect. 2026 Feb 8;29(1):e70590. doi: 10.1111/hex.70590 (PMC12883676; doi:10.1111/hex.70590)
Supplement: Supplementary file 4 — S4: Consolidated Criteria for Reporting Qualitative Research (COREQ): 32‐ item checklist. [file HEX-29-e70590-s001.docx]

**S4 Consolidated Criteria for Reporting Qualitative Research (COREQ): 32-item checklist** (Tong et al., 2007)

Domain 1: Research team and reflexivity

| **Item** | **Description** | **Addressed in manuscript** |
| --- | --- | --- |
| 1 | Interviewer/facilitator | Lead facilitator (LO) identified; facilitation rotated across team (Methods: Workshop process). |
| 2 | Credentials | Research team described as multidisciplinary with expertise in gerontology, health services research, training design, co-design, and knowledge translation (Discussion: strengths). |
| 3 | Occupation | Academic researchers from two universities (Methods: Research team). |
| 4 | Gender | Three female and one male researchers (Methods: Research team). |
| 5 | Experience/training | Co-design and facilitation expertise noted; EBCD toolkit and principles applied (Methods: Design). |
| 6 | Relationship established | Introductory video and PowerPoint provided before workshops to build rapport (Methods: Stage 1 Set-up). |
| 7 | Participant knowledge of researcher | Study purpose and approach explained in consent materials (Methods: Recruitment). |
| 8 | Interviewer characteristics | Reflexive notes on facilitation style, team dynamics, and influence on group process (Methods: Research team). |

Domain 2: Study design

| **Item** | **Description** | **Addressed in manuscript** |
| --- | --- | --- |
| 9 | Methodological orientation | Experience-Based Co-Design (EBCD) explicitly stated (Methods: Design). |
| 10 | Sampling | Purposive recruitment via networks, forums, and social media (Methods: Recruitment). |
| 11 | Method of approach | Invitations by email, flyers, researcher networks (Methods: Recruitment). |
| 12 | Sample size | Seven participants (four providers, three consumers) (Results: Participant characteristics; Table 1). |
| 13 | Non-participation | No dropouts; declining survey completion noted (Results: Co-design process evaluation). |
| 14 | Setting | Online via Zoom workshops (Methods: Workshop process). |
| 15 | Presence of non-participants | None present during workshops (Methods: Workshop process). |
| 16 | Description of sample | Participant demographics reported (Results: Table 1). |
| 17 | Interview guide | Structured run sheets; preparatory video and PPT; communication checklist used (Methods: Stage 1 Set-up). |
| 18 | Repeat interviews | Three iterative workshops plus surveys (Methods: Workshop process). |
| 19 | Recording | Sessions recorded and transcribed (Methods: Data collection). |
| 20 | Field notes | Researcher field notes documented and discussed in debriefs (Methods: Data collection). |
| 21 | Duration | Three one-hour workshops across six weeks (Methods: Workshop process). |
| 22 | Data saturation | Consensus reached using ≥60% threshold; iterative refinement across workshops (Methods: Data collection and analysis). |
| 23 | Transcripts returned | Transcripts not returned; instead, findings validated through surveys and subsequent workshops (Methods: Data collection). |

Domain 3: Analysis and findings

| **Item** | **Description** | **Addressed in manuscript** |
| --- | --- | --- |
| 24 | Number of data coders | Four researchers (RA, LO, LT, ML) analysed data (Methods: Data collection and analysis). |
| 25 | Coding tree | Competencies prioritised and clustered into themes; consensus threshold applied (Methods: Data collection and analysis). |
| 26 | Derivation of themes | Themes derived inductively from transcripts and survey feedback (Methods: Data analysis). |
| 27 | Software | SPSS used for survey analysis; transcripts thematically coded (Methods: Data collection). |
| 28 | Participant checking | Validation occurred across workshops and via evaluation surveys (Results: Final training prototype; Co-design process evaluation). |
| 29 | Quotations presented | Illustrative quotations provided (Results: Table 3). |
| 30 | Data and findings consistent | Findings supported with tables, quotes, and survey results (Results section). |
| 31 | Clarity of major themes | Major themes reported (e.g., core competencies, module structure, evaluation outcomes) (Results: Tables 2–3). |
| 32 | Clarity of minor themes | Sub-competencies and practical considerations (delivery preferences, CPD recognition) presented (Results: Final training prototype). |
