# Supplementary material for: Health Literacy‐Focused Communication Training for Primary Healthcare Providers Working With Older Adults: A Co‐Designed Prototype
Source: Health Expect. 2026 Feb 8;29(1):e70590. doi: 10.1111/hex.70590 (PMC12883676; doi:10.1111/hex.70590)
Supplement: Supplementary file 4 [file HEX-29-e70590-s004.docx]

Pre co-design session 2 survey

Start of Block: Block 3

Q7 Torrens University

Co-designing a Primary Healthcare Provider Communication Training
**Welcome to the Pre-Workshop 2 Survey!**

Thank you for participating in this co-design process. This survey will help us refine and prioritise the key **knowledge, skills, and attitudes** that should be included in a primary healthcare provider communication training program focused on health literacy.

Q2  

We are using the **Knowledge, Skills, and Attitudes (KSA) framework** because it:

	Includes **practical abilities** essential for effective performance and behaviour change
	Is applicable in **professional development** contexts
	Encompasses **adaptability and resilience** in dynamic healthcare environments
	Helps evaluate **competencies, performance, and outcomes**—ideal for curriculum development

Your input is invaluable in shaping this training program.

End of Block: Block 3

Start of Block: Block 1

Q3 A recent study (Beese et al., 2025) provides helpful insights into health literacy-focused communication for healthcare providers, defining it as a two-dimensional construct:

1. Relates to the health professionals themselves and includes their motivation, knowledge and competencies to find, understand, appraise, and apply health-related information to make sound decisions on health and well-being as an individual.

2. Focuses on their interaction with patients, relatives, clients, and healthcare users. It includes their attitudes, knowledge, and skills to promote, maintain, and support the development and practice of health literacy of others (e.g., patients and their relatives).

*"This dualistic understanding leads to new opportunities and challenges in conceptualizing and operationalizing health literacy. Additional competencies are required when addressing the second dimension."* – Beese et al., 2025

**Reference:**

Beese, A. S., Jaks, R., Alder, E., & Gani, S. M. D. (2025). A Delphi study on health literacy competencies for health professionals. BMC Medical Education, 25(1), 38.

| Page Break |  |
| --- | --- |

Q5 Our training program will target interactive and inter-relational core competencies essential for effective provider-patient communication.

The **17 competencies** in this survey have been selected from three sources:

• Recent evidence from the academic literature

• Ideas and priorities from Co-Design Session 1

• Findings from 18 international training interventions

**Your task:** 

• Prioritise each competency based on its importance for the training.

• Determine the best delivery format: online (self-paced, videos, modules, etc.) or in-person (interactive workshops, role-plays, etc.).

**Please complete this survey by Wednesday, March 12.**

Your responses will be compiled and shared in **Co-Design Session 2.**

End of Block: Block 1

Start of Block: Default Question Block

Q1 For each of the **17 competencies** (Knowledge (K), Skills (S), and Attitudes (A)), please:

1️. **Drag and drop** each competency into the delivery method you think is most suitable **(online or in-person).**

2️. **Rank them in order of priority** as you go.

| Competencies that can be effectively taught through self-paced online modules (e.g., videos, interactive content) | Competencies requiring more intensive/in-person approaches (e.g., role-play, simulation, etc.) |
| --- | --- |
| ______ Introduction to Geriatrics (K) (27) | ______ Introduction to Geriatrics (K) (27) |
| ______ Trauma informed communication (K) (28) | ______ Trauma informed communication (K) (28) |
| ______ Interersonal communication (IPC) principles and styles (K) (29) | ______ Interersonal communication (IPC) principles and styles (K) (29) |
| ______ Communicating with patients with limited health literacy (K) (4) | ______ Communicating with patients with limited health literacy (K) (4) |
| ______ Express the attitude that effective communication is essential for ensuring safety and high-quality healthcare (A) (5) | ______ Express the attitude that effective communication is essential for ensuring safety and high-quality healthcare (A) (5) |
| ______ Have the ability to ask patients and their relatives’ prior knowledge about their health problems in an appreciative way (S) (6) | ______ Have the ability to ask patients and their relatives’ prior knowledge about their health problems in an appreciative way (S) (6) |
| ______ Have the ability to create good conditions for discussion (S) (7) | ______ Have the ability to create good conditions for discussion (S) (7) |
| ______ Have the ability to ask effective questions of patients and their relatives using the ‘patient-centred’ approach (ie: prioritise individual needs, shared decision making) (S) (8) | ______ Have the ability to ask effective questions of patients and their relatives using the ‘patient-centred’ approach (ie: prioritise individual needs, shared decision making) (S) (8) |
| ______ Use appropriate methods effectively to assess the understanding of the patient and their relatives (S) (9) | ______ Use appropriate methods effectively to assess the understanding of the patient and their relatives (S) (9) |
| ______ Awareness of unconscious biases (S) (10) | ______ Awareness of unconscious biases (S) (10) |
| ______ Active listening and observation (S) (11) | ______ Active listening and observation (S) (11) |
| ______ Adjusting medical jargon (S) (13) | ______ Adjusting medical jargon (S) (13) |
| ______ Body language awareness (S) (15) | ______ Body language awareness (S) (15) |
| ______ Working with the influence of disinformation (S) (16) | ______ Working with the influence of disinformation (S) (16) |
| ______ Detect patient fibs/withholding (S) (17) | ______ Detect patient fibs/withholding (S) (17) |
| ______ GP preparation (eg: knowing who audience is) (S) (18) | ______ GP preparation (eg: knowing who audience is) (S) (18) |
| ______ Follow up with patients (S) (19) | ______ Follow up with patients (S) (19) |

End of Block: Default Question Block

Start of Block: Block 2

Q7 Are there any other competencies you believe should be included? If so, how should they be delivered (ie: online or in-person)?

________________________________________________________________

________________________________________________________________

________________________________________________________________

________________________________________________________________

________________________________________________________________

Q6 Do you have any comments or suggestions about the competencies?

________________________________________________________________

________________________________________________________________

________________________________________________________________

________________________________________________________________

________________________________________________________________

End of Block: Block 2
